# An Organic Solvent-Tolerant α-L-Rhamnosidase from *Dictyoglomus thermophilum* and Its Application in Production of Icariside I from Icariin

**DOI:** 10.3390/molecules30132847

**Published:** 2025-07-03

**Authors:** Jinyue Hu, Lingling Song, Le Zhao, Xiaoke Zheng, Weisheng Feng, Haoyu Jia

**Affiliations:** 1 School of Pharmacy, Henan University of Chinese Medicine, Zhengzhou 450046, China; 19861108297@163.com (J.H.); 18838221762@163.com (L.S.); zhaole1983@126.com (L.Z.); zhengxk.2006@163.com (X.Z.); fwsh@hactcm.edu.cn (W.F.); 2 The Engineering and Technology Center for Chinese Medicine Development of Henan Province, Zhengzhou 450046, China

**Keywords:** α-L-rhamnosidase, organic solvent-tolerant, enzymatic characterization, icariside I

## Abstract

Icariside I, a bioactive flavonoid derivative derived from *Herba epimedii*, demonstrates better pharmacological properties compared to its precursor icariin. Enzymatic conversion of icariin to icariside I using α-L-rhamnosidase represents an efficient biotechnological approach. In this study, we characterized a GH78 family α-L-rhamnosidase from *Dictyoglomus thermophilum* (*Dth*Rha) with promising biocatalytic properties. The recombinant *Dth*Rha displayed optimal activity at 55 °C and pH 6.0, with remarkable thermostability (retaining > 80% activity after 1 h at 45–65 °C) and pH stability (pH 5.0–7.0). The kinetic parameters *K*_m_, *k*_cat_ and *k*_cat_/*K*_m_ values for pNPR of 0.44 mM, 7.99 s^−1^ and 18.16 s^−1^ mM^−1^, respectively. Notably, *Dth*Rha exhibited good organic solvent tolerance, retaining > 50% activity after 4 h in 10% DMSO. Applied in a DMSO cosolvent system, *Dth*Rha achieved 92.3% conversion of icariin to icariside I within 4 h under optimized conditions. Interestingly, elevating the substrate concentration to 10 mM resulted in a consistently high icariin conversion of 95.8%. The enzymatic hydrolysis method can be applied to the industrial production of Icariside I. Furthermore, *Dth*Rha not only cleaves the α-1,2 glycosidic bond between glucoside and rhamnoside in compounds like naringin, but also exhibits tolerance to organic solvents, making it suitable for the hydrolysis of other poorly soluble flavonoids.

## 1. Introduction

Icariside I and icaritin, two of the primary bioactive compounds derived from *Herba Epimedii*, a traditional Chinese medicinal herb, have garnered significant scientific interest in recent years owing to their diverse pharmacological properties. These compounds exhibit a broad spectrum of biological activities, including anti-inflammatory, anti-cancer, anti-osteoporotic, and immunomodulatory effects [1,2,3,4]. Icaritin has recently gained regulatory approval for the treatment of advanced hepatocellular carcinoma in the form of an icaritin soft capsule [5]. Icariside I exhibits potent immunomodulatory and anti-tumor properties, demonstrating greater therapeutic potential than icaritin for certain diseases, particularly through its ability to reverse tumor immune desertification [6]. Various methods for the production of icariside I have been explored, including extraction from plants, chemical synthesis, and enzymatic transformation. However, direct extraction from plants is significantly limited by the extremely low natural abundance of icariside I (<0.1% in the raw material of *H. Epimedii*), which hinders its large-scale preparation. Chemical synthesis suffers from complex procedures and environmental pollution. In recent years, biocatalysis has garnered increasing attention as a promising alternative due to its advantages of high efficiency, mild reaction conditions and environmental benignity [7].

α-L-rhamnosidase (EC 3.2.1.40) is a well-known hydrolase that can release the terminal L-rhamnose from flavonoid glycosides by cleaving α-1,2, α-1,3, α-1,4, α-1,6, and α-1 glycosidic bond [8]. α-L-rhamnosidase belongs to the GH28, GH78, and GH106 glycoside hydrolase families, among which the GH78 family is the majority. This enzyme is widely distributed across microorganisms, plants, and animals. It has been utilized in various applications, including the reduction of bitterness in citrus fruit juices [9,10], enhancement of wine aroma [11], and facilitation of the synthesis of numerous pharmaceuticals and their precursors [12,13]. Recently, a considerable number of rhamnosidases have been cloned and characterized from various sources, for their potential in the biotransformation of flavonoids. Lou et al. reported a novel α-L-rhamnosidase from *P. laurentii* ZJU-L07, achieving over 87% conversion of epimedin C to icariin, but the large-scale application was limited because this enzyme was an intracellular enzyme [14]. An α-L-rhamnosidase gene from *Thermoclostridium stercorarium* was cloned and expressed, exhibiting the capacity to catalyze the conversion of rutin to isoquercitrin. However, its thermal stability was suboptimal at elevated temperatures, which limits its practical application in high-temperature processes [15]. Cheng et al. identified and characterized a α-L-rhamnosidase from *Talaromyces stollii* CLY-6, which exhibited exceptional hydrolytic activity toward icariin, with a high catalytic efficiency of 179.67 mM^−^^1^ s^−^^1^ [16]. However, its activity rapidly declined when the reaction temperature exceeded 45 °C, indicating a significant loss of stability at higher temperatures. Although the high catalytic conversion rates of these enzymes, substrate concentration remains limited due to the poor solubility of flavonoids in aqueous solutions. To address this, strategies such as increasing the temperature or utilizing biphasic systems can be employed. This requires the enzyme to be tolerant to high temperature and organic solvents, or to employ immobilization to enhance its applicability [17,18,19]. For example, Vila-Real et al. reported a naringinase from *Penicillium decumbens*, the catalytic efficiency was enhanced 10-fold in the biphasic systems containing 3% 1,2-dimethoxyethane [20]. Moreover, both the solubility and bioconversion yield of naringin were further enhanced at higher temperature. As far as we know, there are few reports on α-L-rhamnosidases that are resistant to high temperature and organic solvents.

Herein, we report the successful heterologous expression of an α-L-rhamnosidase (*Dth*Rha) from *Dictyoglomus thermophilum* in *Escherichia coli* BL21 (DE3), followed by comprehensive biochemical characterization. As a representative member of the glycoside hydrolase (GH) 78 family, *Dth*Rha displays high sequence and structural homology with canonical GH78 enzymes, featuring a conserved catalytic module organized into an (α/α)6-barrel fold. Biochemical analyses revealed that *Dt*hRha exhibited better thermostability and remarkable tolerance to organic solvents. The kinetic parameters of *Dth*Rha were investigated, demonstrating its ability to catalyze the conversion of icariin in organic-aqueous system. The α-L-rhamnosidase identified in this study also provides a promising biocatalyst for the scalable and sustainable industrial production of icariside I, as well as other poorly soluble flavonoids.

## 2. Results and Discussion

### 2.1. Sequence and Phylogenetic Analysis

**Figure 1:**
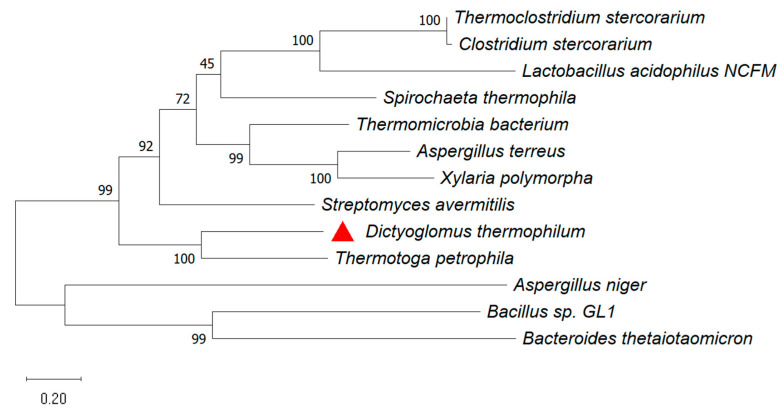
Evolutionary analysis of *Dth*Rha (red triangle in figure) and other GH78 α-L-rhamnosidases using the ML method in MEGA 11 software.

The α-L-rhamnosidase gene *Dth*Rha (GenBank: ACI19983.1) from *Dictyoglomus thermophilum* DSM 3960 is a 2763 bp fragment that encodes a protein of 921 amino acids and belongs to the glycoside hydrolase 78 (GH78) family. A multiple sequence alignment analysis of *Dth*Rha, in comparison with previously characterized α-L-rhamnosidases, is presented in Figure S1. This alignment highlights both conserved regions and variations among these enzymes, providing valuable insights into their structural and functional similarities. Among the analyzed enzymes, *Dth*Rha showed the highest sequence identity with the α-L-rhamnosidase from *Thermotoga petrophila* DSM 13,995 [21] (46.25%) followed by the α-L-rhamnosidase of *Streptomyces avermitilis* MA-4680 [22] (35.47%) and *Spirochaeta thermophila* DSM 6192 [23] (31.32%). Despite the low sequence similarity and domain variability, these enzymes share conserved motifs, specifically IPTDCPQRDERMGWMGDAQL (470–489) and TTLWERWEKL (778–787). The presence of these highly conserved motifs in the catalytic domain is indicative of the α-L-rhamnosidase activity of *Dth*Rha. The phylogenetic trees were constructed using the maximum likelihood (ML) method based on 9 protein sequences, to further investigate the evolutionary relationship between *Dth*Rha and other GH78 α-L-rhamnosidases (Figure 1). The phylogenetic analysis revealed that the α-L-rhamnosidase from *Dictyoglomus thermophilum* and the GH78 α-L-rhamnosidases from *Thermotoga petrophila* DSM 13,995, *Streptomyces avermitilis* MA-4680, and *Thermomicrobia bacterium* PRI-1686 clustered together on the same evolutionary branch. Therefore, the sequence and evolutionary relationship analysis suggested that *Dth*Rha may possess distinct functional properties.

### 2.2. Purification and Characterization of Recombinant DthRha

**Figure 2:**
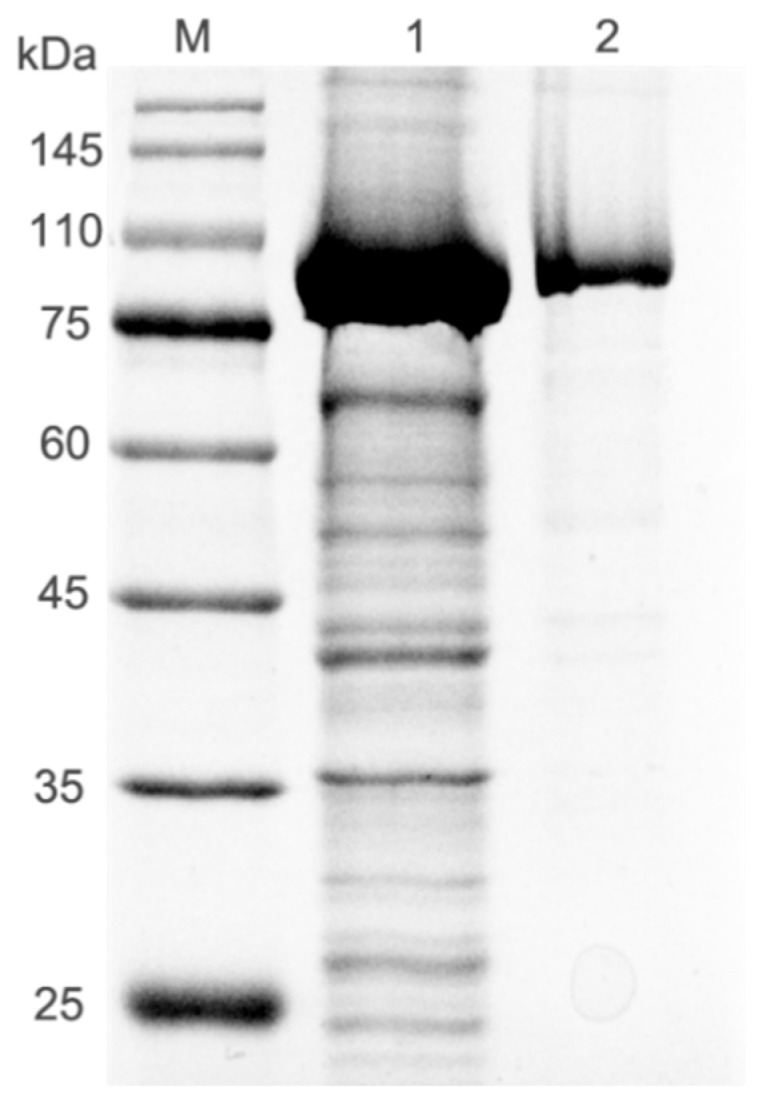
SDS-PAGE analysis of recombinant protein *Dth*Rha. Lane M: protein marker, lane 1: crude extract, lane 2: purified *Dth*Rha.

The induced recombinant pET-28a-*Dth*Rha cells disrupted by sonication and purified by the HisTrap^TM^ HP column to isolate the target protein following the method described previously [24]. The purified *Dth*Rha was identified and analyzed by SDS-PAGE (Figure 2). The molecular weight of the enzyme was approximately 105 kDa without the other bands, which is consistent with the theoretical molecular weight of 106 kDa.

**Figure 3:**
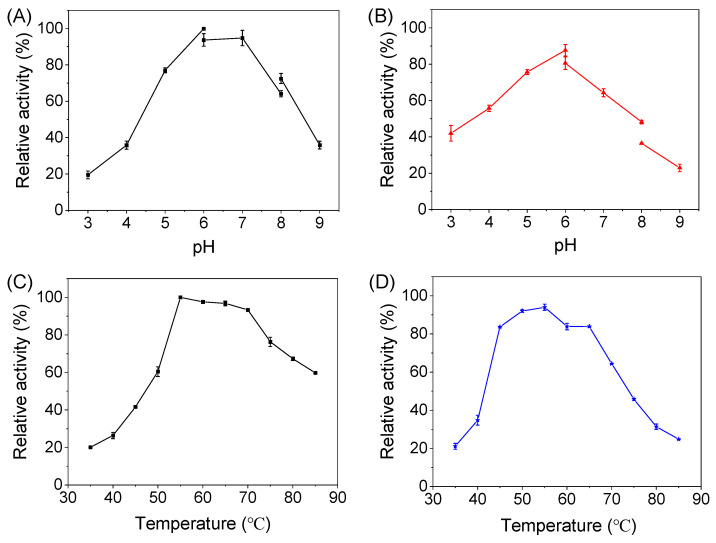
Effects of pH and temperature on the activity and stability of *Dth*Rha. The optimal pH (**A**) and temperature (**C**) of *Dth*Rha. pH (**B**) and thermostability (**D**) stability of *Dth*Rha. Reaction conditions: (**A**) 1 mL of buffer (100 mM) containing 3 mM pNPR, 0.2 mg/mL *Dth*Rha. The optimal pH was assayed at 55 °C, the buffers containing NaAc-HAc buffer (pH 3–6), phosphate buffer (pH 6–8) and Tris-HCl buffer (pH 8–9). (**B**) 0.2 mg/mL *Dth*Rha was incubated in different pH buffers at 4 °C for 24 h to determine pH stability, Subsequently, it was incubated at 55 °C for 5 min, and 3 mM pNPR was added to measure enzyme activity. (**C**) 1 mL of NaAc-HAc buffer (100 mM, pH 6) containing 3 mM pNPR, 0.2 mg/mL *Dth*Rha, at a temperature range of 35–85 °C. (**D**) 0.2 mg/mL *Dth*Rha was incubated at 35–85 °C for 1 h, then, 3 mM pNPR was added to measure the residual activity of *Dth*Rha in NaAc-HAc buffer (100 mM, pH 6) at 55 °C. (**A**,**C**): 100% represents the maximum initial reaction rate (0.9 and 0.83 mM min^−1^) measured in NaAc-HAc buffer (100 mM, pH 6.0). Relative activities at other pH and temperature values were calculated as ratios to this reference value (0.9 and 0.83 mM min^−1^). (**B**,**D**): 100% corresponds to the initial reaction rate at 0 h for each condition, Relative activity after incubation = (Reaction rate after incubation/Initial rate) × 100%.

The enzymatic properties of *Dth*Rha were characterized using pNPR as the substrate. As shown in Figure 3A, the optimal pH for *Dth*Rha was measured to be 6.0. Enzyme activity retained above 70% of its maximum activity within the pH range of 5.0–8.0, indicating that the enzyme is active across a broad pH spectrum. Additionally, the enzyme exhibited good pH stability during storage, retaining more than 70% of its maximum activity at 4 °C for 24 h within a pH range of 5.0–7.0 (Figure 3B). The decline in the activity of *Dth*Rha during storage at 4 °C in different buffers. Which may be attributed to the potential interactions between the storage buffer and the catalytic residues over time (Figure S2), which could alter the local environment of the active site and impair catalytic efficiency [25,26]. The thermal properties of *Dth*Rha were assessed by measuring enzyme activity at various temperatures (35–85 °C). The optimal temperature for *Dth*Rha was found to be 55 °C, lower than the organism’s growth temperature of 78 °C. Investigation revealed that the His-tag of *Dth*Rha had some negative effect on optimal temperature (Figure S3), potentially influencing its structural integrity [27,28]. Although the optimal temperature of this enzyme is slightly lower than that of previously reported α-L-rhamnosidases from *Alternaria alternata* SK37.001 (60 °C) [9], *Aspergillus terreus* CCF3059 (65 °C) [29] and *Lactobacillus plantarum* WCFS1 (70 °C) [30]. It is noteworthy that the enzyme retained over 90% of its maximum activity within the temperature range of 55–70 °C (Figure 3C). Thermostability assays of *Dth*Rha revealed that its residual activity exceeded 80% after incubation at 45–65 °C for 1 h, exhibiting better thermostability (Figure 3D). The results suggest that *Dth*Rha is a suitable catalyst for the hydrolysis of icarrin at high temperatures, where it significantly increases the solubility of icarrin.

The effects of various metal ions and reagents on the activity of *Dth*Rha were examined at final concentrations of 1 mM and 10 mM (Table S1). Most metal ions did not inhibit the activity of *Dth*Rha, with Zn^2+^, Mn^2+^, Fe^2+^, and Fe^3+^ demonstrating a notable activation effect on the enzyme. The relative activities of *Dth*Rha in the presence of Fe^2+^ and Fe^3+^ at a final concentration of 1 mM were approximately 2.04- and 1.54-fold higher, respectively, compared to the enzyme without pre-incubation with these metal ions. Additionally, the reagents DTT and EDTA showed a slight activation effect on *Dth*Rha activity at a concentration of 1 mM, though the effects were not statistically significant.

### 2.3. Effects of Organic Solvents on DthRha Activity

**Figure 4:**
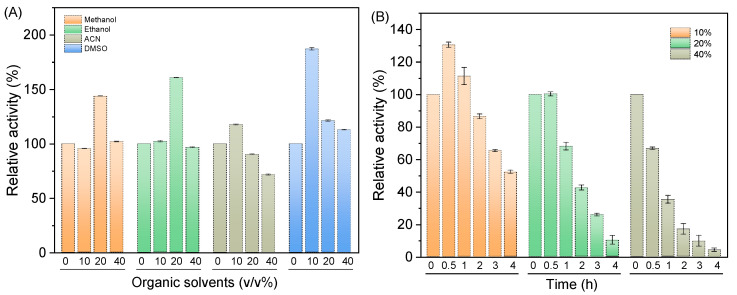
(**A**) Effects of organic solvent on *Dth*Rha activity and (**B**) DMSO stability of *Dth*Rha. Reaction conditions: (**A**) 1 mL of NaAc-HAc buffer (100 mM, pH 6.0) containing 3 mM pNPR, appropriate amount of *Dth*Rha, organic solvents (0–40, *v*/*v*%), mixed and immediately measure the absorbance at 405 nm. (**B**) 1 mL of NaAc-HAc buffer (100 mM, pH 6.0) containing 3 mM pNPR, appropriate amount of *Dth*Rha, organic solvents (10–40, *v*/*v*%), mixed and incubated at 55 °C for different times, then measure the absorbance at 405 nm. The *Dth*Rha activity in the absence of organic solvents (**A**) or at 0 h (**B**) was defined as 100%.

Enzyme stability in the presence of organic solvents is a crucial factor in biocatalysis and industrial applications, where organic solvents are commonly used to improve the solubility of substrates. The effect of different concentrations of organic solvents on *Dth*Rha activity was evaluated, as seen in Figure 4A. *Dth*Rha exhibits strong tolerance to four organic solvents-methanol, ethanol, acetonitrile (ACN), and dimethyl sulfoxide (DMSO)-with its activity remaining above 90% when the solvent concentration is below 20%. Interestingly, *Dth*Rha activity increased by 43.9%, 60.8%, and 87.2% when exposed to 20% methanol, 20% ethanol, and 10% DMSO, respectively. A similar result has been reported for the α-L-rhamnosidase from *Brevundimonas* and *Spirochaeta thermophila* [31,32], where enzyme activity also increased in the presence of organic solvents. In addition, *Dth*Rha retained 42.5% of its initial activity after incubation for 2 h in the buffer containing 20% DMSO, and more than 50% of its initial activity after incubation for 4 h in the buffer containing 10% DMSO, demonstrating better tolerance to DMSO (Figure 4B). DMSO is recognized for its ability to enhance enzymatic activity and its excellent solubilizing properties. It has been evaluated for enzyme catalysis in flavonoid-based reactions [33,34,35], and its application in these reactions remains of significant interest due to its potential to improve reaction efficiency. The comparison of the organic solvent tolerance of *Dth*Rha with α-L-rhamnosidases from other sources, as presented in Table 1, revealed that only Rha-N1 and *Tpe*Rha retained more than 80% of their initial enzyme activity in the presence of 10% DMSO [21,36]. Notably, *Dth*Rha demonstrates superior activity in DMSO as a co-solvent compared to α-L-rhamnosidases from other sources. Furthermore, based on the results in Figure 4A, we evaluated the stability of *Dth*Rha in 20% methanol, 20% ethanol, and 10% ACN, as presented in Figure S4. The results indicated that *Dth*Rha retained more than 60% of its initial activity after 2 h of incubation in these solvents. This enhanced solvent tolerance suggests that *Dth*Rha holds significant potential for industrial applications, particularly in the catalytic conversion of icariin to icariside I.

### 2.4. Kinetic Parameters of DthRha

The kinetic parameters of *Dth*Rha, as outlined in Table 2, reveal notable characteristics that differentiate it from other GH78 family α-L-rhamnosidases, with pNPR being used as the substrate. The enzyme exhibited a *K*_m_ value of 0.44 mM, which is considerably lower than that reported for α-L-rhamnosidases from other sources, suggesting a higher substrate affinity. This lower *K*_m_ value signifies a stronger enzyme-substrate interaction, which likely contributes to the enhanced catalytic performance observed for *Dth*Rha. Additionally, *Dth*Rha demonstrated a catalytic efficiency of 7.99 s^−1^, highlighting its better enzymatic activity. These results emphasize the potential advantages of *Dth*Rha in industrial and biotechnological applications, where both high substrate affinity and efficient catalysis are essential for optimal performance.

### 2.5. Substrate Specificity of DthRha

**Figure 5:**
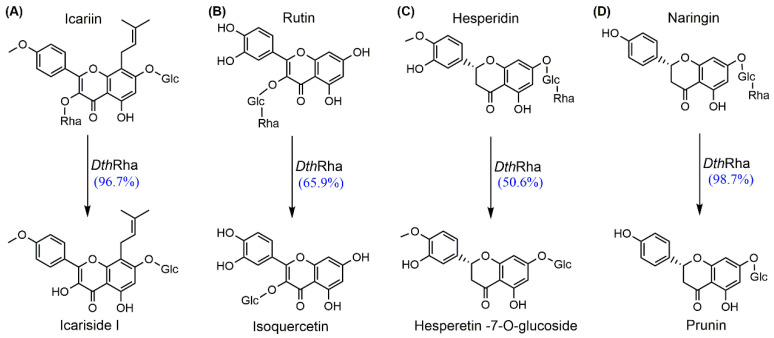
Substrate spectrum of *Dth*Rha (values in parentheses are substrate conversion). (**A**–**D**) *Dth*Rha catalyzed the conversion of icarrin, rutin, hesperidin, and naringin, respectively. Reaction conditions: 2 mL of NaAc-HAc buffer (100 mM, pH 6.0) containing 3 mM substrate, appropriate amount of *Dth*Rha was incubated at 55 °C, 180 rpm.

To determine the substrate specificity of *Dth*Rha, the results are summarized in Table S2. *Dth*Rha exhibited the highest specific activity with pNPR (80.15 U/mg), followed by icariin (5.14 U/mg), naringin (5.01 U/mg), rutin (2.17 U/mg), hesperidin (1.93 U/mg). In addition, we performed enzymatic transformation with icariin, rutin, hesperidin, and naringin as substrates to assess its catalytic efficiency. The results, as shown in Figure 5, provided further insight into the enzyme’s substrate specificity and catalytic performance. *Dth*Rha showed the highest hydrolytic activity toward icariin and naringin, achieving conversion of 96.7% and 98.7%, respectively. This catalytic performance was similar to the α-L-rhamnosidases from *Spirochaeta thermophila* and *Thermotoga petrophila* [32,42]. Furthermore, enzymatic characterization revealed a distinct substrate preference profile for *Dth*Rha, with conversion rates decreasing in the following order naringin (α-1,2) > icariin (α-1) > rutin (α-1,6) > hesperidin (α-1,6). Moreover, a comparison of the initial reaction rates for the four substrates suggested that naringin and icariin may be more suitable substrates for *Dth*Rha, as it exhibited higher catalytic activity towards these compared to rutin and hesperidin (Table S3). Substrate specificity analysis demonstrates that *Dth*Rha functions as a robust and versatile biocatalyst, exhibiting particularly high catalytic activity toward α-1,2 and α-1 rhamnosidic bond.

### 2.6. Enzymatic Production of Icariside I

**Figure 6:**
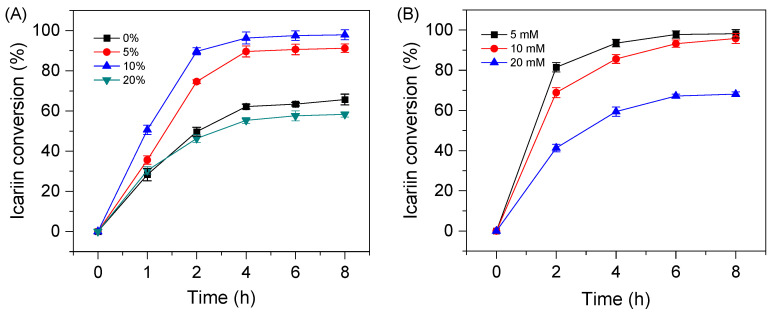
Effect of DMSO concentration (**A**) and substrate concentration (**B**) on the hydrolysis of icariin to icariside I. Reaction conditions: (**A**), 2 mL of NaAc-HAc buffer (100 mM, pH 6.0) containing 3 mM icariin, appropriate amount of *Dth*Rha, DMSO (0–20%, *v*/*v*%) was incubated at 55 °C, 180 rpm. (**B**), 2 mL of NaAc-HAc buffer (100 mM, pH 6.0) containing 5–20 mM icariin, appropriate amount of *Dth*Rha, 10% DMSO was incubated at 55 °C, 180 rpm.

Based on the above research, the enzymatic reaction conditions of icariin I to icaritin by *Dth*Rha need to continue to be investigated. The effect of varying *Dth*Rha concentrations, ranging from 1 to 6 mg/mL, on the enzymatic conversion of icariin to icariside I was investigated. As shown in Figure S5, the conversion of icariin nearly reached its maximum when the enzyme concentration was 4 mg/mL or higher. Based on these results, the optimal enzyme concentration for the conversion of icariin to icariside I was determined to be 4 mg/mL. To further enhance the catalytic efficiency, the concentration of DMSO in the reaction system was optimized. As shown in Figure 6A, the conversion of icariin reached 92.3% when 10% DMSO was used as a cosolvent for 4 h, which was approximately 1.5-fold higher than in the aqueous system. This increase in conversion can be attributed to the enhanced activity of *Dth*Rha and the improved solubility of the substrate in the DMSO cosolvent. However, at a DMSO concentration of 20%, the conversion of icariin decreased, likely due to disruption of enzyme-substrate interactions and partial denaturation of *Dth*Rha caused by the high solvent concentration. Despite this reduction, the conversion of icariin in the 20% DMSO cosolvent system still reached 50.3%. These findings suggest that *Dth*Rha performs more efficiently in the DMSO cosolvent system, making it a more suitable medium for the conversion of icariin to icariside I compared to the aqueous system.

To evaluate the potential of *Dth*Rha for industrial applications, we examined its catalytic performance under high substrate loading conditions in 10% DMSO cosolvent system. The enzymatic hydrolysis of icariin (5 mM) to icariside I was completed within 6 h (Figure 6B). When the substrate concentration was 10 mM, *Dth*Rha achieved a conversion of 95.8% for icariin after 8 h of reaction. However, at higher substrate concentration (20 mM), the conversion decreased significantly to 68.1% over the same duration, suggesting potential substrate inhibition effects that may impair enzyme activity at elevated concentrations.

## 3. Materials and Methods

### 3.1. Strains, Growth Media and Regents

The gene of α-L-rhamnosidase from *Dictyoglomus thermophilum* (*Dth*Rha, GenBank accession: WP_012548615) was codon-optimized, synthesized and cloned into the vector pET-28a by Genscript (Nanjing, China). Competent cells of *E. coli* BL21 (DE3) and *E. coli* DH5α were purchased from TransGen Biotech (Beijing, China). TlANpure Mini Plasmid Kit was purchased from TIANGEN Biotech (Beijing, China). The recombinant strain was cultured in Luria-Bertani (LB) medium containing kanamycin at 37 °C, 180 rpm. Icariin, icariside I and p-nitrophenyl-α-L-rhamnopyranoside (pNPR) was obtained from Bidepharm (Shanghai, China). Kanamycin was obtained from Macklin (Shanghai, China). Restriction endonucleases and T4 DNA ligase were purchased from Thermo Fisher Scientific (Schwerte, Germany). All chemicals were purchased from commercial sources and directly used.

### 3.2. Sequence Analysis of DthRha

The gene sequence of *Dth*Rha, along with other known α-L-rhamnosidase genes from various organisms, was subjected to multiple sequence alignment and the construction of evolutionary trees using the maximum likelihood (mL), all performed with the software MEGA 11.

### 3.3. Expression of DthRha in E. coli BL21 (DE3) and Enzyme Purification

According to previous report, the recombinant plasmids pET-28a harboring *Dth*Rha were transformed into *E. coli* BL21 (DE3) [24]. The recombinant strain *E. coli* pET-28a-*Dth*Rha was cultured in LB medium containing 50 μg/mL kanamycin at 37 °C and 180 rpm until the OD600 reached 0.6–0.8. Subsequently, IPTG was added to a final concentration of 0.1 mM, and the culture was incubated at 20 °C with shaking at 180 rpm for 20 h.

The cells were harvested by centrifugation and resuspended in binding buffer (pH 7.4, 20 mM sodium phosphate, 500 mM NaCl, 10 mM imidazole). Cells disruption was carried out using ultrasonic breaking (30% power, 2 s on, 3 s off, 15 min total at 4 °C). After centrifugation at 4 °C, the supernatant was loaded onto a HisTrap™ HP column (5 mL) equilibrated with binding buffer. The target protein was eluted using elution buffer (pH 7.4, 20 mM sodium phosphate, 500 mM NaCl, 500 mM imidazole). The purified enzyme was desalted using a HisTrap™ Desalting column (5 mL) with desalting buffer (pH 7.4, 20 mM sodium phosphate). The purification of *Dth*Rha without His-tag was carried out as follows: Cell disruption was performed using ultrasonic treatment. Following centrifugation at 4 °C, the supernatant underwent heat treatment (30 min at 50 °C) to remove non-thermostable proteins. After final centrifugation for 20 min, the supernatant was used for biochemical characterization. Protein purity was analyzed by SDS-PAGE and visualized using an image analysis system (Bio-Rad, Hercules, CA, USA).

### 3.4. Assay of Enzyme Activity and Protein Concentration

The activity of *Dth*Rha was determined based on the increase in p-nitrophenol concentration during the hydrolysis of pNPR. As described in our previous report [18], the enzyme assay was performed by mixing 440 μL NaAc-HAc buffer (100 mM, pH 6.0), 40 μL pNPR, and 20 μL *Dth*Rha in a 1.5 mL EP tube. The enzyme activity was actually measured by monitoring the change in absorbance during the first 5 min of the reaction, with absorbance values recorded at 0.5-min intervals. The absorbance of supernatant at 405 nm was measured using a UV-vis spectrophotometer. One unit (U) of enzyme activity was defined as the amount of enzyme required to produce 1 μmoL of product per minute under standard assay conditions. The protein concentration was determined using the Bradford method [43].

### 3.5. Biochemical Characterization of DthRha

The biochemical properties of *Dth*Rha were determined using *p*-NPR as substrate. The effect of pH on *Dth*Rha activity was assessed across a range of pH values (3–9) using NaAc-HAc buffer (100 mM, pH 3–6), phosphate buffer (100 mM, pH 6–8) and Tris-HCl buffer (pH 8–9). The pH stability was assessed by measuring the residual activity of *Dth*Rha after 24 h of incubation at 4 °C across different pH values (3–9). The temperature effect on *Dth*Rha was investigated by measuring the activity in NaAc-HAc buffer (100 mM, pH 6) over a temperature range of 35–85 °C. Thermostability was evaluated by determining the residual enzyme activity following 1 h of incubation at temperatures ranging from 35 to 85 °C. The activities were measured to use relative activity by taking the optimal activity of the enzyme as 100%.

The effect of metal ions and regents on *Dth*Rha activity were studied in the presence of 1 mM or 10 mM chemicals including Na^+^, K^+^, Ca^2+^, Cu^2+^, Mg^2+^, Zn^2+^, Mn^2+^, Fe^2+^, Fe^3+^, Co^2+^, dithiothreitol (DTT) and ethylene diamine tetraacetic acid (EDTA). To evaluate organic solvents tolerance of *Dth*Rha, the residual enzyme activity was measured in the presence of methanol, ethanol, DMSO, and acetonitrile (ACN) (the final concentrations of 10%, 20%, 30%, *v*/*v*). The enzyme activity without metal ions, regents and organic solvents was defined as 100%. The substrate specificity of *Dth*Rha was determined using rutin, hesperidin, naringin, and icariin, with a substrate concentration of 3 mM. The initial reaction rates of *Dth*Rha catalyzing the conversion of the four substrates were evaluated at varying substrate concentrations, ranging from 0.1 to 2 mM, and maintaining a constant enzyme concentration.

### 3.6. Catalytic Kinetic Parameters

The apparent kinetic parameters (*K*_m_ and V_max_) of *Dth*Rha were assessed at 55 °C and pH 6.0 by measuring the initial reaction rates at different pNPR concentrations (0.1–5 mM). The Michaelis-Menten constant (*K*_m_) and maximal reaction rate (V_max_) were obtained by nonlinear fitting of the Michaelis-Menten equation using Origin 2021 software. The catalytic constant (*k*_cat_) was calculated using the equation: *k*_cat_ = V_max_/[E], where [E] represents the total enzyme concentration in the reaction system.

### 3.7. Enzymatic Hydrolysis of Icariin to Icariside I by DthRha

The time course of the enzymatic hydrolysis of icariin to icariside I was monitored by tracking the conversion of icariin. In a typical assay, 2 mL of NaAc-HAc buffer (100 mM, pH 6.0) containing 3 mM icariin and 0.5 U/mL purified *Dth*Rha was incubated at 55 °C with shaking at 180 rpm. At 0.5 h intervals, aliquots were collected from the reaction mixture, diluted with the mobile phase, passed through a 0.22 μm filter membrane after centrifugation, and then analyzed by HPLC. The effects of DMSO concentrations (up to 20% *v*/*v*) and icariin concentrations (5–20 mM) on the conversion of icariin were investigated. The conversion was defined as the percentage of the consumed substrate amount in the initial substrate amount. All experiments were performed in duplicate.

### 3.8. Analytical Methods

The quantitative analysis of flavonoid compounds was conducted using a ZORBAX Eclipse XDB-C18 column (4.6 × 250 mm, 5 μm, Agilent, Santa Clara, CA, USA) on a reversed-phase HPLC system (Shimadzu, LC-16AT, Kyoto, Japan) and eluting with acetonitrile (A) and 0.1% formic acid water (B) at 1 mL/min, with a gradient program: 0–12 min, 28% A–28% A; 12–17 min, 28% A–90% A; 17–18 min, 90% A–90% A; 18–22 min, 90% A–28% A; 22–28 min, 28% A–28% A. The column temperature was maintained at 35 °C, 10 μL of the sample was injected and detected at 270 nm.

## 4. Conclusions

This study presents the biochemical characterization of a GH78 family α-L-rhamnosidase from *Dictyoglomus thermophilum* (*Dth*Rha), which displays low sequence homology with known α-L-rhamnosidases. *Dth*Rha demonstrated better thermostability and remarkable tolerance to organic solvents, along with significantly higher substrate affinity than other reported α-L-rhamnosidases. Notably, when applied in a DMSO cosolvent system, *Dth*Rha efficiently catalyzed the conversion of icariin to icariside I, achieving a significant conversion of 92.3% under optimized conditions (10% DMSO, 4 h). Following optimization of reaction parameters, the enzymatic conversion of 10 mM icariin to icariside I, with a corresponding conversion of 95.8%. These findings establish *Dth*Rha as a promising biocatalyst for the efficient production of icariside I, offering significant advantages in terms of catalytic efficiency and operational stability in organic-aqueous systems.

## Figures and Tables

**Table 1:** Comparison of the organic solvent tolerance of *Dth*Rha with α-L-rhamnosidase from other GH78 family members.

Organism	Enzyme	Organic Solvent (*v*/*v*%)	Residual Activity	Refs.
*Dictyoglomus thermophilum*	*Dth*Rha	DMSO (10%)	86.5%	This study *
*Spirochaeta thermophila*	*St-*Rha	DMSO (10%)	Ca. 67%	[32]
*Thermotoga petrophila*	*Tpe*Rha	DMSO (10%)	82.1%	[21]
*Aspergillus terreus*	*At*Rha	DMSO (10%)	44.6	[37]
*Thermoclostridium stercorarium*	*Tst*Rha	DMSO (10%)	Ca. 61%	[15]
*Paenibacillus odorifer*	*Pod*Rha	DMSO (10%)	Ca. 53%	[38]
*Aspergillus niger*	N12-Rha	DMSO (10%)	Ca. 71%	[39]
*Aspergillus niger*	Rha-N1	DMSO (10%)	Ca. 85%	[36]

* Reaction condition: 1 mL of NaAc-HAc buffer (100 mM, pH 6.0) containing 3 mM pNPR, appropriate amount of *Dth*Rha, DMSO (10%, *v*/*v*), mixed and incubated at 55 °C, measure the absorbance at 405 nm. The Rha residual activity in the absence of organic solvents was defined as 100%.

**Table 2:** Comparison of kinetic parameters of *Dth*Rha with α-L-rhamnosidase from other sources.

Enzyme	*K*_m_ (mM)	*k*_cat_ (s^−1^)	*k*_cat_/*K*_m_ (s^−1^ mM^−1^)	Refs.
*Dth*Rha	0.44	7.99	18.16	This study *
Rha-N1	2.80	0.95	0.339	[36]
*An*Rha	2.9	29	10	[40]
*Bb*Rha	2.2	2.5	1.14	[41]
*Pg*Rha	1.13	43.65	38.6	[12]
*Tpe*Rha	2.99	651.37	219.83	[21]

The kinetic constants of *Dth*Rha and the other enzymes cited above were determined using pNPR as the substrate. * Reaction conditions: 1 mL of NaAc-HAc buffer (100 mM, pH 6.0) containing (0.1–5 mM). 0.2 mg/mL (1.905 μM) *Dth*Rha.

## Data Availability

The original contributions presented in this study are included in the article/Supplementary Materials. Further inquiries can be directed to the corresponding author.

## Supplementary Materials

The following supporting information can be downloaded at: https://www.mdpi.com/article/10.3390/molecules30132847/s1, Figure S1: Sequences comparison between DthRha and other α-L-rhamnosidase through multi-alignment. Figure S2: Stability of *Dth*Rha in aqueous buffer storage and lyophilized powder storage. Figure S3: The effect of His-tag on the optimal temperature and thermostability of *Dth*Rha. Table S1: Effects of metal ions and reagents on the activity of *Dth*Rha; Figure S4: Stability of *Dth*Rha in Methanol, Ethanol and ACN. Table S2: Substrate specificity of *Dth*Rha. Table S3: The initial reaction rates of *Dth*Rha catalyzing the conversion of the four substrates. Figure S5: Effect of enzyme concentration on the hydrolysis of icariin to icariside I; Figure S6: HPLC chromatogram of the enzymatic hydrolysis reaction of icariin.
