# Supplementary material for: An Organic Solvent-Tolerant α-L-Rhamnosidase from Dictyoglomus thermophilum and Its Application in Production of Icariside I from Icariin
Source: Molecules. 2025 Jul 3;30(13):2847. doi: 10.3390/molecules30132847 (PMC12251106; doi:10.3390/molecules30132847)
Supplement: Supplementary file 1 [file molecules-30-02847-s001.zip › molecules-3655045-supplementary.pdf]

Supplementary Files for Article

An Organic Solvent-Tolerant α-L-Rhamnosidase from *Dictyoglomus thermophilum* and Its Application in Production of Icariside I from Icariin

Jinyue Hu [1], Lingling Song [1], Le Zhao [1], Xiaoke Zheng [1,2], Weisheng Feng [1,2] and Haoyu Jia [1,*]

1 School of Pharmacy, Henan University of Chinese Medicine, Zhengzhou 450046, China; 19861108297@163.com (J.H.); 18838221762@163.com (L.S.); zhaole1983@126.com (L.Z.); zhengxk.2006@163.com (X.Z.); fwsh@hactcm.edu.cn (W.F.)

2 The Engineering and Technology Center for Chinese Medicine Development of Henan Province, Zhengzhou 450046, China

* Correspondence: jiahaoyu@hactcm.edu.cn

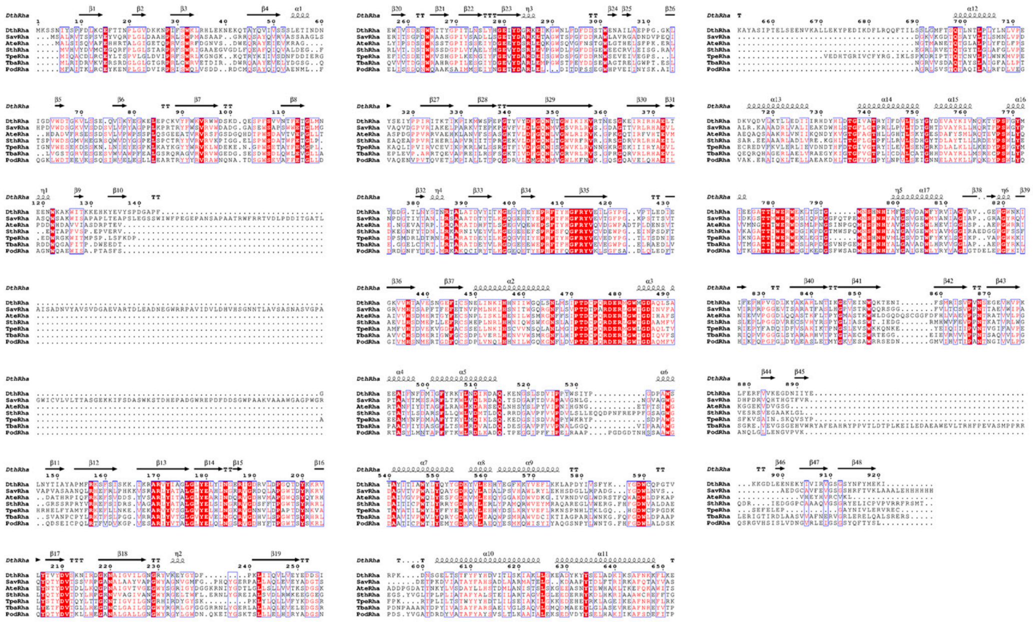

Figure S1. Sequences comparison between *Dth*Rha and other α-L-rhamnosidase through multi-alignment. *Dictyoglomus thermophilum* (*Dth*Rha), *Streptomyces avermitilis* MA-4680 (*Sav*Rha), *Aspergillus terreus* CCF 3059 (*Ate*Rha), *Spirochaeta thermophila* DSM 6192 (*St*hRha), *Thermotoga petrophila* DSM 13995 (*Tpe*Rha), *Thermomicrobia bacterium* (*Tba*Rha), *Paenibacillus odorifer* (*Pod*Rha).

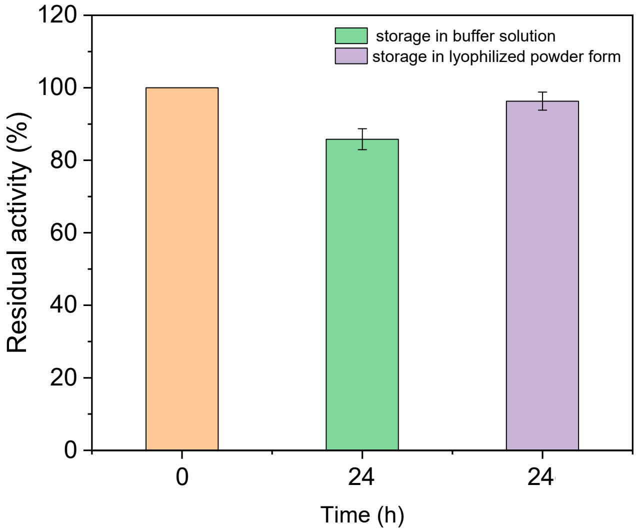

Figure S2. Stability of *Dth*Rha in aqueous buffer storage and lyophilized powder storage. Conditions: Aqueous buffer storage: The lyophilized powder was reconstituted in NaAc-HAc buffer (100 mM, pH 6.0), and both the reconstituted sample and the lyophilized powder were stored at 4 °C for 24 h. The activity of the lyophilized powder of *Dth*Rha at 0 h was defined as 100%.

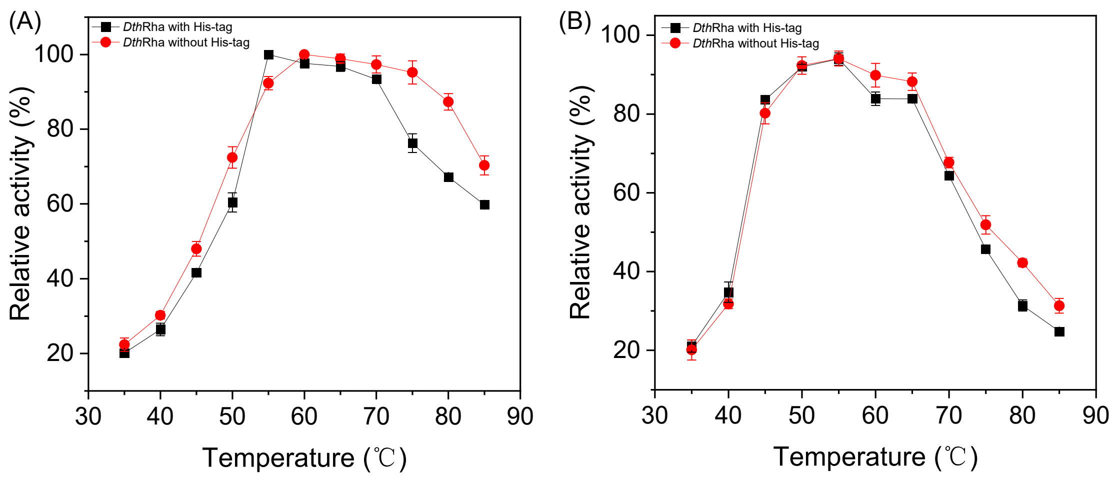

Figure S3. The effect of His-tag on the optimal temperature and thermostability of *Dth*Rha. Reaction conditions: (A) 1 mL of NaAc-HAc buffer (100 mM, pH 6) containing 3 mM pNPR, 0.2 mg/mL DthRha, at a temperature range of 35-85 °C. (B) 0.2 mg/mL DthRha was incubated at 35-85 °C for 1 h, then, 3 mM pNPR was added to measure the residual activity of DthRha in NaAc-HAc buffer (100 mM, pH 6) at 55 °C. (A): the maximum enzyme activity was defined as 100%. (B): the initial activity at 0 h was defined as 100%, the conditions are the same as in Figure 3.

Table S1. Effects of metal ions and reagents on the activity of *Dth*Rha

Cation of reagent*	Relative activity (Mean%±SD)	
	1 mM	10mM
Contrl	100.0±1.5	100.0±1.3
Na^{+}	87.6±2.7	91.8±1.3
K^{+}	115.6±1.3	102.7±0.8
Ca^{2+}	139.2±0.6	118.6±1.5
Cu^{2+}	121.0±2.2	101.2±0.5
Mg^{2+}	138.6±0.1	90.4±2.4
Zn^{2+}	149.4±0.7	159.3±1.0
Mn^{2+}	129.7±1.3	144.5±1.4
Fe^{2+}	204.6±0.4	202.2±1.9
Fe^{3+}	154.0±1.3	178.0±1.1
Co^{2+}	124.3±0.9	177.7±0.6
DTT	121.8±1.1	93.1±3.1
EDTA	126.6±0.3	124.8±2.1

*Final concentration, 1 mM and 10 mM as indicated. Data represents the means of duplicate experiments, and the SD represents the standard deviation.

Reaction conditions: 1 mL of NaAc-HAc buffer (100 mM, pH 6.0) containing 3 mM pNPR, appropriate amount of *Dth*Rha, 1 mM or 10 mM metal ions, incubated at 55 °C, measured the absorbance at 405 nm. The relative activity in the absence of metal ions was defined as 100%.

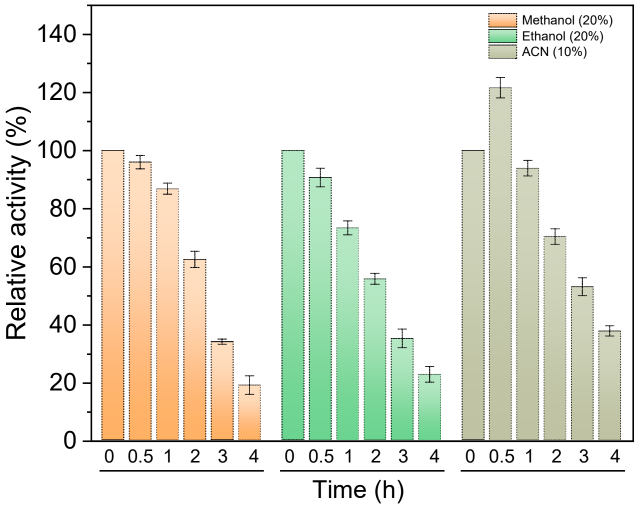

Figure S4. Stability of *Dth*Rha in Methanol, Ethanol and ACN.

Reaction conditions: 1 mL of NaAc-HAc buffer (100 mM, pH 6.0) containing 3 mM pNPR, appropriate amount of *Dth*Rha, organic solvents (10-20, v/v%), mixed and incubated at 55°C for different times, then measure the absorbance at 405 nm. The *Dth*Rha activity at 0 h was defined as 100%.

Table S2. Substrate specificity of *Dth*Rha.

Entry	Substrate	Product	Specific activity (U/mg)
1	pNPR	p-nitrophenol	80.15
2	Naringin	Prunin	5.01
3	Icariin	Icariside I	5.14
4	Rutin	Isoquercetin	2.17
5	Hesperidin	Hesperetin-7-O-glucoside	1.93

One unit of *Dth*Rha was defined as the amount of enzyme required to produce 1 μmol of product per minute.

Table S3. The initial reaction rates of *Dth*Rha catalyzing the conversion of the four substrates.

Entry	Substrate	Product	Initial reaction rates (V_0, μM s^{-1})
1	Naringin	Prunin	1.07
2	Icariin	Icariside I	1.02
3	Rutin	Isoquercetin	0.66
5	Hesperidin	Hesperetin-7-O-glucoside	0.68

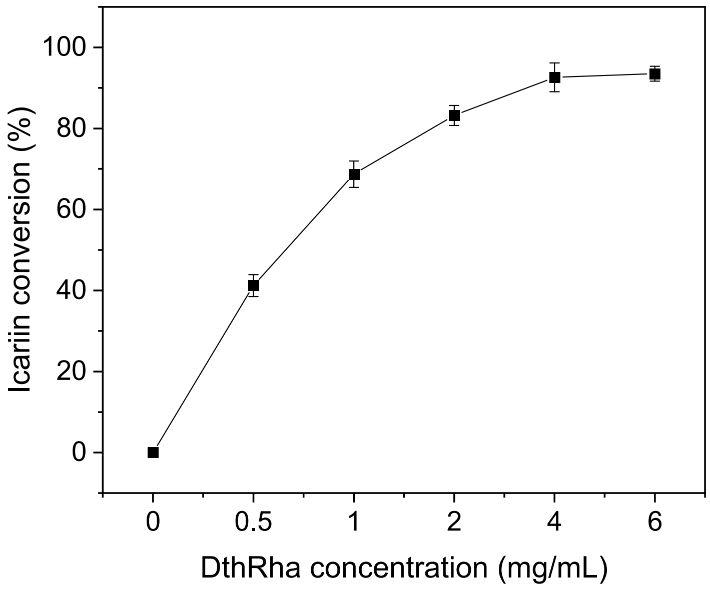

Figure S5. Effect of enzyme concentration on the hydrolysis of icariin to icariside I.

Reaction conditions: 2 mL of NaAc-HAc buffer (100 mM, pH 6.0) containing 3 mM icariin and 0-6mg/mL purified *Dth*Rha was incubated at 55 °C with shaking at 180 rpm.

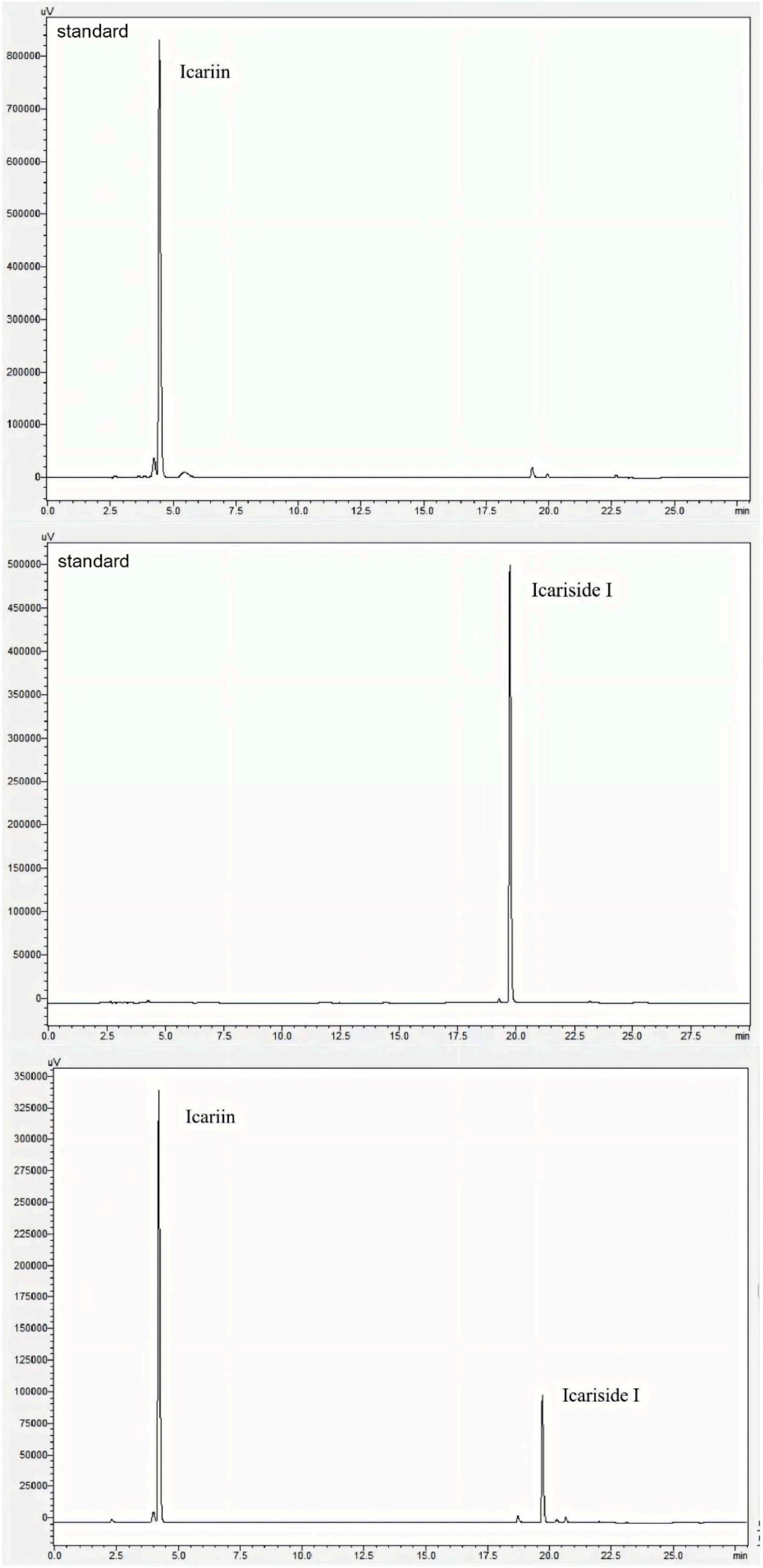

Figure S6. HPLC chromatogram of the enzymatic hydrolysis reaction of icariin.
